# Regulation of bone and fat balance by Fructus Ligustri Lucidi in ovariectomized mice

**DOI:** 10.1080/13880209.2023.2168019

**Published:** 2023-02-05

**Authors:** Xiaoyan Qin, Qiu Wei, Ran An, Yun Yang, Mingqi Cai, Xiaoling Han, Haoping Mao, Xiumei Gao

**Affiliations:** a Key Laboratory of Pharmacology of Traditional Chinese Medical Formulae, Ministry of Education, Tianjin University of Traditional Chinese Medicine, Tianjin, China; b Tianjin State Key Laboratory of Modern Chinese Medicine, Tianjin University of Traditional Chinese Medicine, Tianjin, China

**Keywords:** Ovariectomy, osteoblastogenesis, adipogenesis, postmenopausal osteoporosis (PMOP), bone marrow mesenchymal stem cells (BMMSCs)

## Abstract

**Context:**

Fructus Ligustri Lucidi (FLL), a commonly used herb of traditional Chinese medicine (TCM), is the fruit of *Ligustrum lucidum* Ait. (Oleaceae). The ethanol extract of FLL is a potential candidate for preventing and treating postmenopausal osteoporosis (PMOP) by nourishing the liver and kidneys.

**Objective:**

This study determines whether an ethanol extract of FLL has anti-osteoporotic effects in ovariectomized (OVX) mice and explores the underlying mechanism.

**Materials and methods:**

The OVX model of eight-week-old C57BL/6J female mice was taken, and ovariectomy was used as PMOP. Mice were divided into five groups: sham-operated group (*n* = 10), OVX group (*n* = 10), OVX + E_2_ group (*n* = 10; 0.039 mg/kg), OVX + FLL group (*n* = 10; 2 g/kg) and OVX + FLL group (*n* = 10; 4 g/kg). Mice were treated by gavage with FLL or CMCNa once daily for 8 weeks. We harvested uteri, femur, and tibias from mice; bone mineral density (BMD) and bone microstructure were obtained by X-ray absorptiometry and micro-CT. Furthermore, the effect of FLL on the balance of osteoblast and adipocyte differentiation was investigated using bone marrow mesenchymal stem cells (BMMSCs).

**Results:**

The results indicated that FLL did not affect OVX-induced estradiol reduction. Compared with OVX mice, FLL significantly increased BMD (63.54 vs. 61.96), Conn. D (86.46 vs. 57.00), and left tibial strength (13.91 vs. 11.27), decreased Tb. Sp (0.38 vs. 0.44) and body fat content (4.19% vs. 11.24%). FLL decreased osteoclast activity and enhanced RUNX2 expression; inhibited perilipin peroxisome proliferator-activated receptor gamma (PPARγ) expression and adipocyte differentiation from BMMSCs.

**Conclusions:**

FLL prevented additional bone loss and improved bone microstructure in OVX mice by modulating bone and fat balance, suggesting that FLL might be a therapeutic agent for PMOP.

## Introduction

Menopause is a natural part of aging in women transitioning from a reproductive to a non-reproductive status. Due to the dramatic changes after menopause, some common metabolic diseases, such as osteoporosis and obesity, occur in postmenopausal women. Among postmenopausal diseases, osteoporosis severely affects the quality of life of postmenopausal women, as osteoporosis-related fractures are the leading cause of disability and even death in older women. It is estimated that nearly half of postmenopausal women will experience an osteoporotic fracture (Eastell and Rosen 2019). On the other hand, obesity and increased numbers of bone marrow fat cells are associated with osteoporosis in menopausal women. Despite our previous conventional reasoning that obesity protects against osteoporotic fractures because peripheral obesity provides insulation during falls, the belief that obesity protects against osteoporosis has recently been revised (Greco et al. 2015). Excessive intra-abdominal fat, visceral obesity, or yellow fat in the marrow have been reported to be associated with osteoporosis (Kim et al. 2003), and postmenopausal women with increased risk of PMOP with visceral obesity and excess intra-abdominal fat (van Gemert et al. 2019; Yuasa et al. 2020). Hence, the reciprocal regulation between bone and adipose tissue may provide a potential means to study the underlying mechanisms of PMOP and develop drugs that can be used for postmenopausal osteoporosis (PMOP).

Bone remodeling is a process that consists of bone resorption by osteoclast and bone formation by osteoblast. Osteoclasts can be differentiated from bone marrow monocytes under the effect of M-CSF and RANKL. Bone marrow mesenchymal stem cells (BMMSCs) are another kind of important cells that are responsible for the formation of bone tissue and the maintenance of bone homeostasis. Bone marrow-derived BMMSCs constitute the pluripotent cell population in the bone marrow (Georgiou et al. 2012; Kim et al. 2021). Under certain conditions, BMMSCs differentiate into various cells, such as osteoblasts, adipocytes, and chondrocytes (Ji et al. 2020). RUNX2 and PPARγ are two canonical markers of osteogenic and adipogenic differentiation from BMMSCs, respectively. It represents a typical reciprocal relationship in bone marrow (Jang et al. 2020). When adipocyte differentiation increases, there is a corresponding decrease in osteoblast differentiation; this reduces the ability to form bone and disrupts the balance between osteoblasts and osteoclasts, resulting in decreased bone mineral and bone mass (Griffith et al. 2006; Yu et al. 2018). Therefore, it is critical to maintain bone health by effectively inhibiting the differentiation of BMMSCs into adipocytes.

Shennong’s Herbal Classic describes Fructus Ligustri Lucidi (FLL) as a method of nourishing the yin and nourishing the kidney. It is a commonly used herbal remedy for age-related diseases (Siu et al. 2013). According to the National Health Commission of the People’s Republic of China, FLL is a traditional Chinese medicine (TCM) that can be used in nutraceuticals (Wei and Jian 2002). It is reported that FLL extract has multiple therapeutic properties, such as antitumor activity, hepatoprotective, immunomodulatory, anti-inflammatory, amelioration of hyperlipidemia (Gao et al. 2015). FLL extract affects bone turnover and calcium balance in ovariectomized rats (Zhang et al. 2006; Dong et al. 2012). It exerts anti-osteoporotic effect in corticosterone-induced osteoporosis (Bian et al. 2011) and oxidative stress-related osteoporosis (Wu et al. 2021). FLL also inhibits adipogenesis in ovariectomized mice (Liu et al. 2022). The previous work of our group found that the Erzhi formula (composed of *Eclipta prostrata* L and FLL in a ratio of 1:1) significantly inhibited bone loss and body weight gain induced by OVX in mice (Qin et al. 2021). However, whether FLL used alone can regulate bone loss and weight gain in ovariectomized mice is still unknown. Therefore, the current study was performed to elucidate the effect and its underlying mechanisms of FLL on bone loss and weight gain in ovariectomized mice. Then bone related cells such as osteoclast induced from monocytes and the balance of BMMSCs differentiation between osteoblast and adipocytes were examined in the present study that might afford a potential herbal medicine in prevention of PMOP and obesity in post-menopausal women.

## Materials and methods

### Preparation of FLL extracts

FLL was purchased from Hebei Anguo Medicinal Materials Market and certified by Tianjin University of Traditional Chinese Medicine (No. FLL 20161229). To prepare the FLL extract, 500 g of crude FLL was extracted twice with 5000 mL of 80% ethanol in a reflux unit for 2 h each. After refluxing, the extract was evaporated in a rotary evaporator and lyophilized in a freeze-dryer. The final extraction rate of FLL was 27.3%. The identification and quality control of FLL extracts were described in our previous work (Pang et al. 2018; Wang et al. 2020).

### Animals

Eight-week-old female C57BL/6 mice weighing 20–22 g were purchased from Beijing Weitong Lihua Experimental Animal Technology Co., Ltd., certificate number SCXX (Beijing) 2016-0006. Mice were housed under standard specific pathogen-free (SPF) conditions at the Institute of Radiation Medicine, Chinese Academy of Medical Sciences at 22 °C–25 °C, 58 ∼ 65% relative humidity, and exposed to a 12-h light/dark cycle; Provide standard food pellets and water *ad libitum*. The Animal Ethics Committee approved Tianjin University of Traditional Chinese Medicine (ethical approval number: TCM-LAEC2021007) in accordance with NIH (United States) guidelines (NIH publication #85-23, revised 1985).

After one week, 50 female mice were divided into a sham-operated group (*n* = 10) and ovariectomized group (OVX) (*n* = 40). After one week, plasma estradiol levels were significantly reduced, confirming the success of OVX. One week after the operation, the mice were randomly divided into four groups: OVX group (0.2% carboxymethyl cellulose sodium (CMC-Na), *n* = 10), E_2_ group (0.039 mg/kg conjugated estrogens tablets produced by Xinjiang Xinziyuan Bio-Pharmaceutical Co., Ltd.; H20090172, *n* = 10), 2 g/kg FLL group (2 g raw FLL/kg body weight, *n* = 10), and 4 g/kg FLL group (4 g raw FLL/kg body weight, *n* = 10). The choice of FLL dose was based on the clinical dose recommendations in Chinese Pharmacopeia 2020. We also observed that the dose of FLL was almost similar to other studies (Tang et al. 2018; Chen et al. 2021). All the animals were dosed by gavage for eight weeks.

### Body weight and food intake

We recorded the body weight and food intake of all animals every 3–4 d for eight consecutive weeks after the operation.

### Analysis of body composition

One day before the sacrifice, animals were placed in a test tube under isoflurane anesthesia, and their weights were recorded. A nuclear magnetic resonance body composition analyzer (QMR06-090H, Suzhou Niumai Analytical Instrument Co., Ltd.) was used to determine body composition, including body fat and lean mass.

### Uterine morphology in OVX mice

After eight weeks of treatment with FLL, mice were sacrificed, and uteruses were removed for weighing and photographic examination.

### Enzyme-linked immunosorbent (ELISA) assay

According to the manufacturer’s instructions, plasma estradiol concentrations were determined using a kit purchased from R&D Systems (USA). Plasma markers of bone turnover include tartrate-resistant acid phosphatase (Trap), tartrate-resistant acid phosphatase 5 b (Trap5b), osteoprotegerin (OPG), and receptor activator of nuclear factor kappa-B ligand (RANKL) were measured with ELISA kits purchased from CUSABIO (China).

### Bone mineral density and microstructure

After sacrifice, the left femur was collected and cleaned by removing the attached muscle. Bone mineral density in the left femur was determined by dual-energy X-ray absorptiometry with an InAlyzer instrument (Faxitron Bioptics, USA). The bone microstructure was analyzed by a micro-computed tomography (micro-CT) system (VivaCT40, SCANCO micro-CT, Switzerland). A 3 mm scan was performed on the femur metaphysis of the mouse. Moreover, morphometric analysis was started, with the first slice located at 1 mm from the metaphyseal line in 100 continuous slices. Based on micro-CT images in specific orientations, 3D images were constructed, and trabecular bone parameters were analyzed.

### Left tibial strength

The strength of the left tibia was measured with a small animal bone strength tester (YLS-16A, China). After cleaning the surrounding muscles, place the tibia horizontally in the instrument and click the start button. This value will be displayed after the bone is broken.

### Primary culture of bone marrow mesenchymal stem cells

Rinse the bone marrow of the bilateral femur and tibia with phosphate-buffered saline (PBS) containing 10% penicillin-streptomycin. After filtration through a 70 µm filter, the filtrate was centrifuged at 300 g for 5 min. Then cells were collected and treated with α-MEM (Gibco; Thermo Fisher Scientific) without phenol red containing 10% fetal bovine serum (FBS, BI), 100 U/mL penicillin, and 100 μg/mL streptomycin (BI). Cells were incubated at 37 °C under 5% CO_2_. The medium was changed after 48 h, followed by a fresh medium every 2 d. The third-generation BMMSCS were used for the experiments.

### Adipogenic differentiation in vitro

The third-generation BMMSCs were seeded in each well of a 96-well plate at a concentration of 10^5^ cells/mL. Adipogenic differentiation medium (containing 0.1 mM dexamethasone, 0.5 mM IBMX, 10 µg/mL insulin, and 200 µM indomethacin) was added for 14 d, and cells were then finally stained with Oil Red O (Solarbio, G1260). Cells in the control group were treated only with a medium without adipogenic differentiation medium or herbs. The experiment was repeated at least three times.

### Osteoblast differentiation in vitro

The third generation of BMMSCs was seeded in each well of a 96-well plate at a concentration of 5 × 10^4^ cells/mL. Osteoblast differentiation medium (containing 0.1 µm dexamethasone, 10 mM sodium β-glycerophosphate, and 50 µM vitamin C) was added for seven days, followed by alkaline phosphatase (ALP) with BCIP/NBT as a chromogenic substrate (Beyotime, C3206). The experiment was repeated at least three times.

### Osteoclast culture

The cells were derived from 4-week-old C57BL/6 female mice. The bone marrow of bilateral femurs and tibias was flushed with PBS containing 10% penicillin–streptomycin solution, mononuclear cells were isolated with a sterile Ficoll solution, and red blood cells were removed using a red blood cell lysis buffer. Cells were maintained in α-MEM supplemented with 10% FBS (BI), penicillin (100 U/mL), and streptomycin (100 µg/mL, BI). Incubations were performed at 37 °C in 5% CO_2_. The cells were seeded in a 96-well plate at 3 × 10^5^ cells/mL for 24 h. Osteoclast differentiation was then induced using macrophage colony-stimulating factor (M-CSF, 2.5 µg/mL) and receptor activator of NF-kB ligand (RANKL, 5 µg/mL). Change the medium every three days. Dissolve FLL in 0.1% dimethyl sulfoxide and diluted to appropriate concentrations (0.01, 0.1, and 1 µg/mL) with the α-MEM medium. The experiment was repeated at least three times.

### Trap-activity assay and trap staining

Trap activity was used as a marker of osteoclast differentiation. Cells were seeded in a 96-well plate and induced into osteoclasts. After 5 d of culture, the medium in the plate was transferred to a new plate for the Trap-activity assay using a Trap Assay Kit (Beyotime). Cells were washed twice with PBS and fixed with 4% paraformaldehyde for 30 min; we followed the Trap-staining kit manufacturer’s protocol (Sigma–Aldrich). Trap-positive multinuclear cells were identified as containing more than three nuclei that appeared dark red. The experiment was repeated at least three times.

### F-actin staining

After fixing the cells with 4% paraformaldehyde for 30 min, we permeabilized the cells with 0.1% Triton X-100 for 10 min. Cells were washed twice with PBS and sealed with 1% bovine serum albumin (BSA) for 30 min. Cells were washed twice with PBS again, Alexa Fluor 568 phalloidin (1:50) as a high-affinity F-actin probe was added at 40 µL/well, and cells were then incubated at 37 °C for 40 min. The Hoechst stain was added for 15 min, and the cells were kept in the dark. F-actin ring formation was measured using an inverted fluorescence microscope (Zeiss, Germany). The experiment was repeated at least three times.

### Real-time PCR

Total RNA was extracted with Trizol reagent (Invitrogen Life Technology, Carlsbad, CA), and 1 µg of RNA was reversed and transcribed into cDNA. Real-time PCR was performed using the FastStart Universal SYBR Green Master (ROX) mix (Roche, Germany), and amplification was completed using the ABI Prism 7300 Sequence Detection System. The amplification conditions were as follows: initial 2 min at 95 °C and 40 cycles of denaturation at 95 °C for 15 s, annealing at 65 °C for 60 s, and extension at 72 °C for 15 s. Results were calculated using the 2^−ΔΔC^_t_ method. The primer sequences are shown in Table 1.

**Table 1.** Names and sequences of primers used for polymerase chain reaction analysis.

Gene	Sequence
GAPDH	F: 5′GGTCGGAGTCAACGGATTTGG3′, R: 5′CTCCTGGAAGATGGTGATGGG3′
PPARγ	F: 5′GGGTAAGCTCTTGTGAATGG3′ R: 5′CTGATGCACTGCCTATGAGC3′
Pref-1	F: 5′CCTGGCTGTGTCAATGGAGT3′
	R: 5′CAAGTTCCATTGTTGGCGCA3′
Zfp-423	F: 5′CGCGATCGGTGAAAGTTGAA3′
	R: 5′CGATCACACTCTGGCTCTCC3′
ACP5	F: 5′CGATGCCAGCGACAAGAGGTTC3′
	R: 5′CTGTGCAGAGACTTGCCAAGG3′
NFATC1	F: 5′ACCACCAGCCACGAGATCATCC3′
	R: 5′AACTCGGAAGACCAGCCTCACC3′
Runx2	F: 5′-GCGTCAACACCATCATTCTG-3′
	R: 5′-CAGACCAGCAGCACTCCATC-3′

### Western immunoblotting analysis

Extract proteins from bone marrow using a 1 mL syringe containing PBS, and add red blood cell (RBC) lysis buffer (RIPA, Solarbio) to lyse RBCs (PMSF = 100:1; Solarbio, R0020, P0100) on ice for 10 min. Total protein was extracted directly from cells using a lysis buffer. Next, centrifuge the suspension at 12,000 g for 10 min at 4 °C, and the supernatant was collected into a new centrifuge tube for later use. The manufacturer’s protocol used the Pierce Rapid Gold BCA Protein Assay Kit (Thermo Fisher, A53225) to determine total protein concentration. The target protein expression was detected using SDS-PAGE (40 µg total protein per well), electrophoresed at 50 V for 30 min and 100 V for 1 h. Then the protein was transferred to the PVDF membrane (Millipore). The membranes were blocked with 5% nonfat dry milk in TBST (0.5% Tween in TBS) for two hours and then incubated overnight at 4 °C with rabbit polyclonal antibody diluted 1:1000 with TBST (RUNX2, Abcam, ab76056; OPG, Abcam, ab203061; RANKL, Absin, abs120177; PPARγ, Affinity, AF6284). The next day, the membranes were washed three times for 5 min with TBST and incubated with an appropriate secondary antibody (ZB2301, 1:10,000) at room temperature for 2 h. The protein bands were visualized using ECL western blotting substrate (P90719, Millipore) and exposed with a ChemiDoc MP Imaging System (BIO-RAD, 734BR4251). The protein expression level was defined with the gel imaging analysis system ImageJ (National Institutes of Health, USA) and normalized with the corresponding β-actin or GAPDH as the internal control.

### Dual-luciferase reporter assay

293T cells were co-transfected with TOPflash and FOPflash plasmids in 96-well plates (0.36 µg/well) using Lipofectamine 2000 (Thermo Fisher Scientific) without penicillin–streptomycin solution and Renilla plasmid (0.04 µg/well) was co-transferred as an internal reference. Six hours after transfection, 0.01, 0.1, and 1 µg/mL FLL were added to cells. After 24 h of incubation, the Dual-Luciferase Reporter Assay System (Promega) was used to study luciferase activity.

### Statistical analysis

Data are expressed as mean ± standard deviation (SD) and analyzed using IBM SPSS Statistics for Windows (version 22, SPSS Inc., Chicago, IL, USA) and constructed graphs using GraphPad Prism (version 7.0 for Windows, GraphPad Software, LaJolla, California USA). Differences between the two groups were assessed using Student’s *t*-test. When ≥3 groups were explicitly compared, we used one-way ANOVA. Differences with a *p* value of less than 0.05 were considered statistically significant.

## Results

### FLL inhibits OVX-induced uterine atrophy

**Figure 1.**
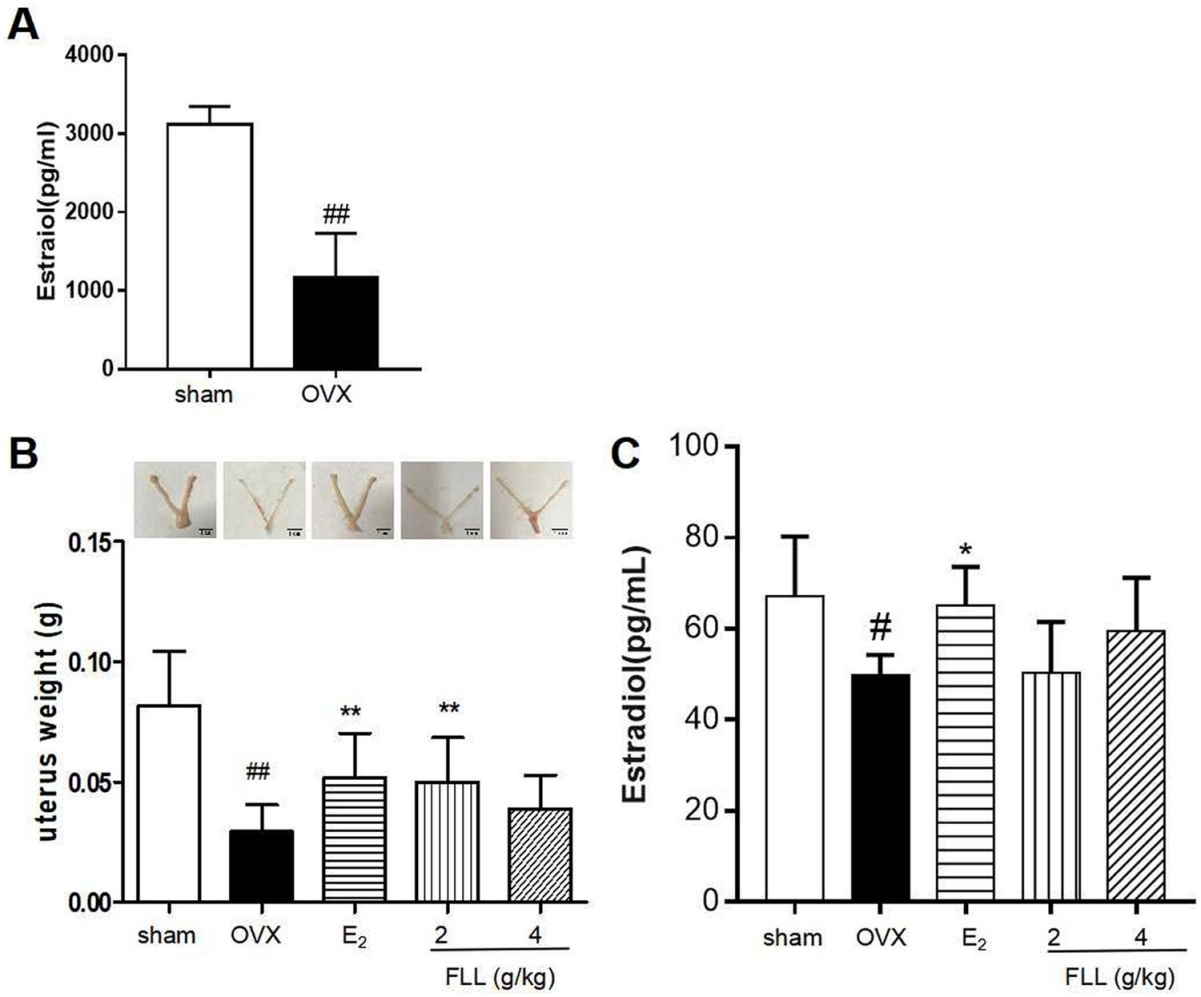
FLL suppresses uterine atrophy induced by OVX. (A) Estradiol concentrations were significantly diminished one week after OVX in all mice as detected by the ELISA kit. (B) Uterine atrophy in OVX mice after treatment with FLL at eight weeks. (C) The level of estradiol after treatment with FLL in OVX mice. ^##^*p* < 0.01 compared with sham; *p < 0.05 compared OVX (*n* = 10).

We successfully established an OVX model by measuring plasma estradiol levels. Our results showed that estradiol levels were significantly reduced one week after OVX in all mice, indicating a successful surgery (*p*** **<** **0.01; Figure 1(A)). Our results also showed that OVX mice had significant uterine atrophy, and the uterine weight of the model group was significantly reduced (*p*** **<** **0.01). E_2_ and FLL treatment reversed uterine atrophy and significantly increased uterine weight (*p*** **<** **0.05; Figure 1(B)). In addition, we detected the serum estradiol levels after FLL treatment, and the results showed that estradiol levels were significantly decreased in OVX mice, but significantly increased by conjugated estrogen tablet treatment. However, estradiol levels did not increase in OVX mice treated with FLL, indicating that the effect of FLL on PMOP was not primarily dependent on estrogen-like effects (*p*** **<** **0.05; Figure 1(C)).

### FLL inhibits bone loss and reduces fat mass in OVX mice

**Figure 2.**
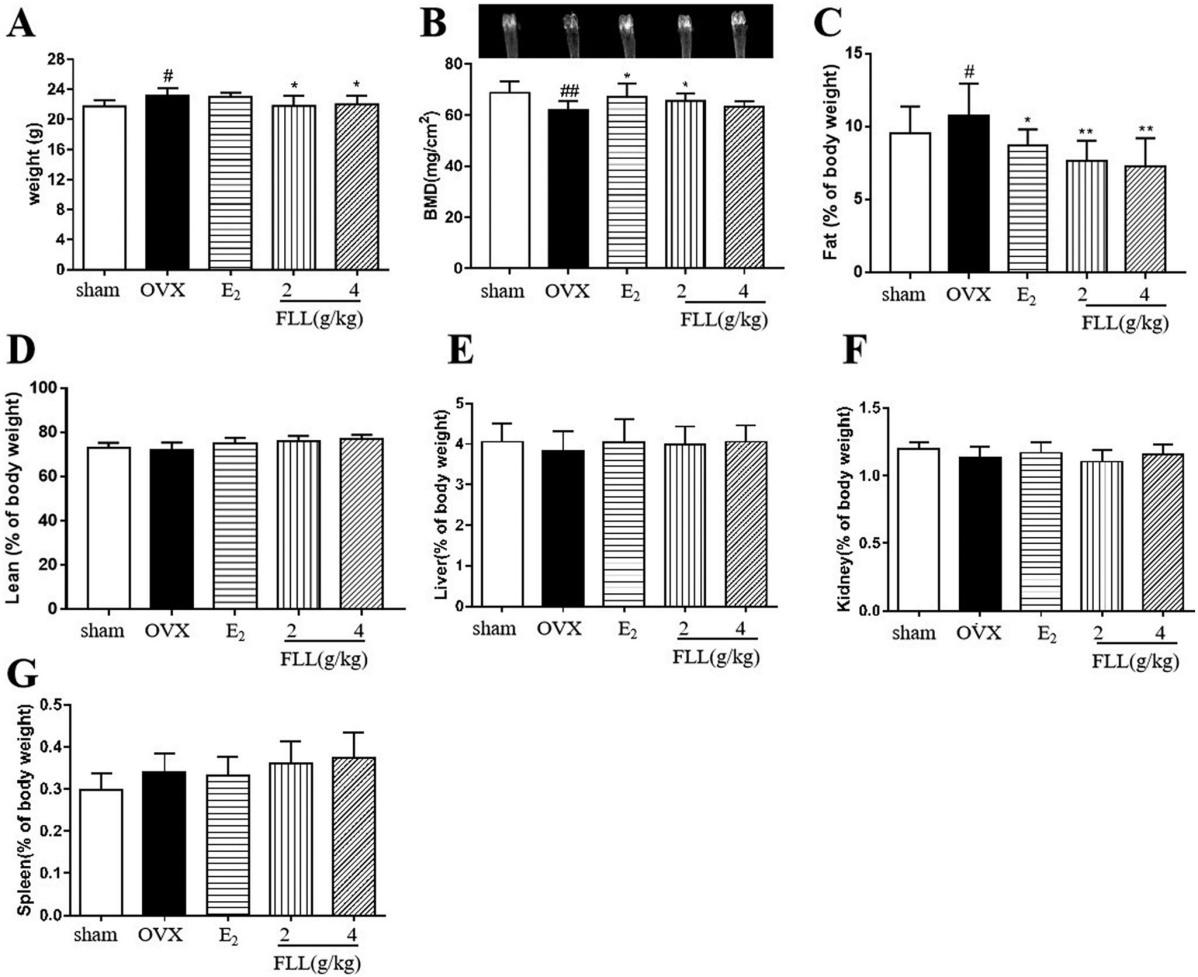
FLL suppresses bone loss and reduces fat weight in OVX mice. (A) Body weights of OVX mice eight weeks post-operatively. Treatment with FLL decreased body weight significantly. (B) Bone mineral density (BMD) in the femurs of OVX mice decreased significantly compared to the sham group, and estrogen and FLL significantly augmented BMD in OVX mice. (C,D) The body fat and lean content of each group were evaluated by an NMR body composition analyzer after eight weeks. (E–G) The indices for liver, kidney, and spleen among groups. ^#^*p* < 0.05 compared with sham; ^##^*p* < 0.01 compared with sham; *p < 0.05 compared OVX; **p < 0.01 compared OVX (*n* = 10).

Our data revealed that OVX significantly increased body weights in OVX mice at the end of the study, whereas treatment with FLL significantly decreased body weight gain (*p*** **<** **0.05; Figure 2(A)); and the changes in body weight were independent of daily diet (*p*** **>** **0.05; Table 2). We also measured the weight of various organs in the mice treated with FLL for eight weeks, including bone, adipose tissue, lean meat, liver, spleen, and kidney. The results of dual-energy X-ray absorptiometry showed that BMD in the femurs of OVX mice was significantly reduced compared with the sham-operated group (*p*** **<** **0.01; Figure 2(B)). E_2_ and FLL (2 g/kg) significantly increased BMD in OVX mice (*p*** **<** **0.01 or 0.05; Figure 2(B)). An NMR body-composition analyzer measured body fat and lean muscle content in each group. The results showed that eight weeks after OVX surgery, OVX mice had increased fat content, whereas E_2_ and FLL groups had significantly lower fat content compared with OVX groups (*p*** **<** **0.01 or 0.05; Figure 2(C)). There were no differences in lean meat content between groups (Figure 2(D)), and we found no differences in the liver, kidney, and spleen indices between groups (Figure 2(E–G)).

**Table 2.** The daily diet of mice.

Groups	Days	Daily diet(*g*)
Sham	60	2.80 ± 0.43
OVX	60	2.78 ± 0.36
E_2_	60	2.78 ± 0.49
FLL (2g/kg)	60	2.97 ± 0.53
FLL (4g/kg)	60	2.75 ± 0.38

### FLL improves bone microstructure and mechanical properties and reduces bone marrow fat content in OVX mice

**Figure 3.**
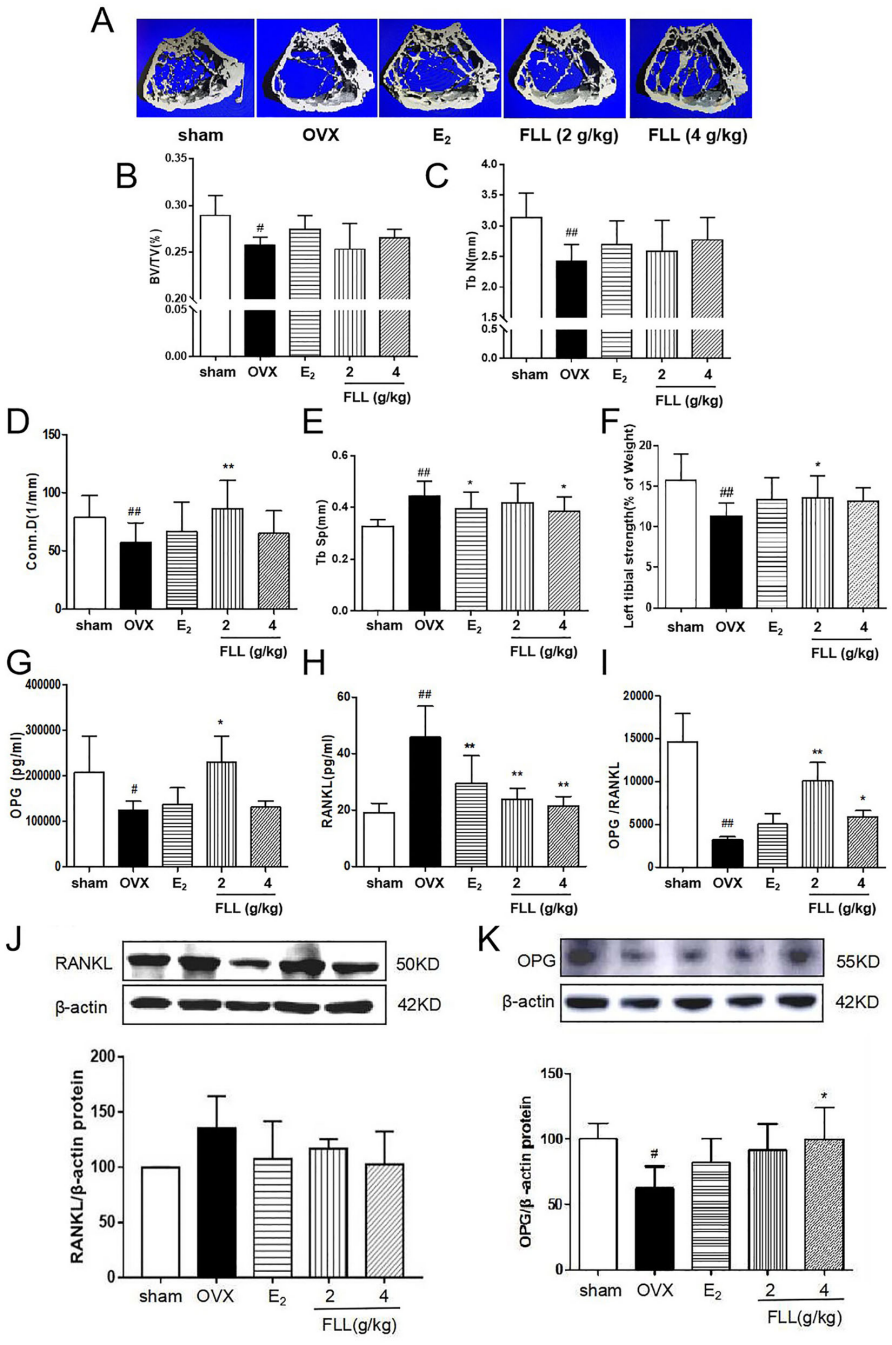
FLL improves bone microstructure and mechanical properties in OVX mice. (A) The microstructure of the distal femur by micro-CT showed that trabecular bone was severely reduced in the model group but recovered with estrogen and FLL treatment. (B,C) The BV/TV and Tb.N were significantly reduction in OVX mice compared with sham but it showed a trend effect of FLL. (D–F) The Conn. D, Tb. Sp and the left tibial strength in the model group were significantly lower than in the sham group but recovered with 2 or 4 g/kg of FLL. (G,H) Plasma levels of OPG and RANKL were measured by ELISA. The level of OPG significantly increased in the FLL group compared to the OVX group, and the level of RANKL was commensurately attenuated. (I) The ratio of OPG/RANKL was significantly reduced in the OVX group, while it significantly increased after treatment with FLL (*n* = 10). (J, K) FLL treatment promoted OPG and inhibited RANKL proteins in bone tissue. ^#^*p* < 0.05 compared with sham; ^##^*p* < 0.01 compared with sham; *p < 0.05 compared with OVX; **p < 0.01 compared with OVX (*n* = 10).

**Figure 4.**
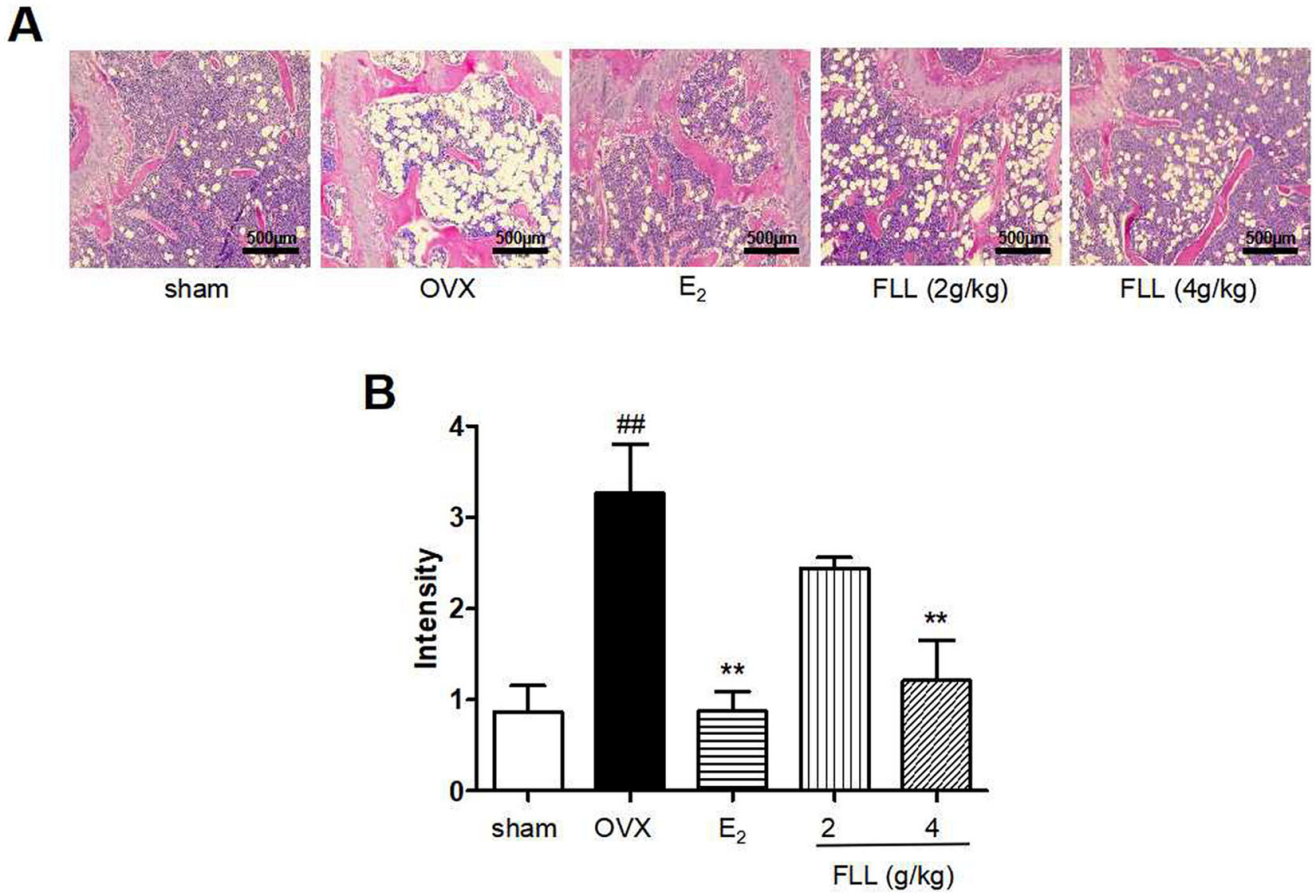
FLL reduces fat content in the bone marrow of OVX mice. (A) H&E staining showed that the white vacuoles in bone marrow increased in the OVX group, while estrogen and FLL significantly reduced the area of white vacuoles after eight weeks of treatment in OVX mice. (B) The white vacuoles were measured quantitatively and data are shown below. ^##^*p* < 0.01 compared with sham; **p < 0.01 compared with OVX (*n* = 3).

As FLL significantly suppressed bone loss in OVX mice, we evaluated the microstructure of the distal femur by micro-CT (Figure 3(A)). Microstructural parameters of trabecular bone in the distal femur showed that BV/TV (Bone volume/Tissue volume) and Tb. N (Trabecular number) was significantly decreased in OVX mice, but FLL showed only a trend effect (Figure 3(B,C)). Conn.D (bone trabecular connection density) was significantly lower in the OVX group than in the sham group (*p*** **<** **0.01); however, FLL (2 g/kg) reversed the change of Conn.D induced by OVX (*p*** **<** **0.01 or 0.05; Figure 3(D)). The Tb. Sp (Trabecular separation) was remarkably higher in OVX mice compared with the sham group, followed by Tb. Sp was significantly decreased after treatment with E_2_ or FLL (4 g/kg) (*p*** **<** **0.01 or 0.05; Figure 3(E)). The strength of the left tibial was significantly decreased in the OVX group compared with that in the sham group (*p*** **<** **0.01), but was dramatically increased in the FLL group (2 g/kg) compared with that in the OVX group (*p*** **<** **0.05; Figure 3(F)). OPG plays a crucial role in preventing osteoporosis, and RANKL binds to OPG, inhibiting the formation of osteoclasts by disrupting RANKL-RANK signaling. The level of OPG in the FLL group was significantly higher than the OVX group (*p*** **<** **0.05). Furthermore, FLL treatment correspondingly suppressed the level of RANKL (*p*** **<** **0.01; Figure 3(G,H)). The ratio of OPG/RANKL was significantly reduced in the OVX group, whereas it was significantly elevated after treatment with FLL (*p*** **<** **0.01; Figure 3(I)). The results for OPG and RANKL proteins in bone tissue were consistent with the plasma results (Figure 3(J,K)). Using H&E staining of femurs, we also found that the adipose tissue that we observed as white vacuoles in bone marrow increased in the OVX group, while E_2_ and FLL significantly reduced the area of white vacuolation after eight weeks of treatment in OVX mice (*p*** **<** **0.01; Figure 4(A,B)).

### FLL inhibits osteoclastogenesis and bone resorption by downregulating Trap activity and Trap5b and NFATc1 levels

**Figure 5.**
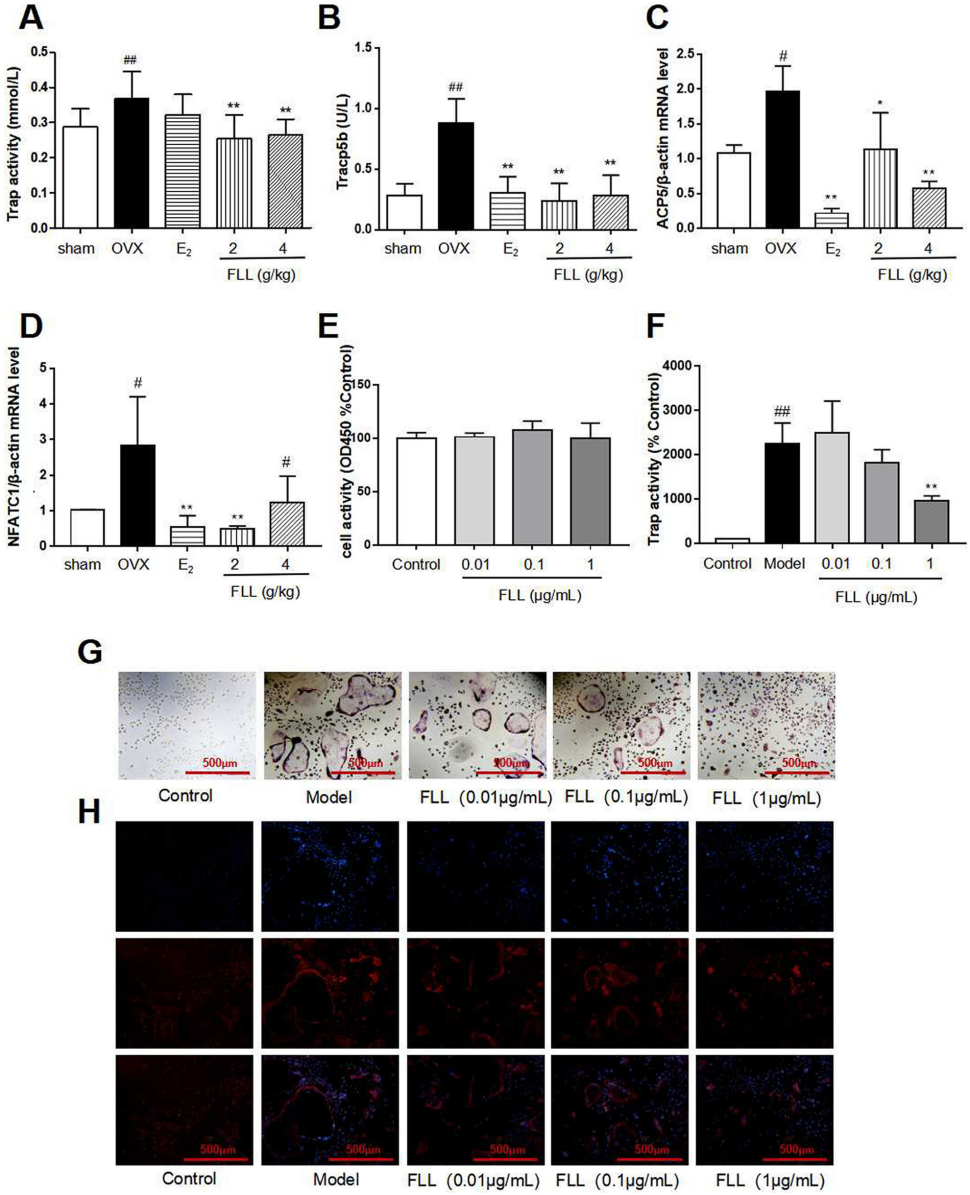
FLL inhibits osteoclastogenesis and function by downregulation of Trap activity and levels of Trap5b and NFATc1. (A,B) The levels of Trap activity and Trap-5b proteins in plasma markedly decreased after treatment with FLL (*n* = 10). (C,D) Trap mRNA expression in the OVX group was significantly higher than in the sham group. Estrogen and FLL suppressed the increase in ACP5 and NFATc1 mRNA expression. (*n* = 3). (E) Monocytes from bone marrow were cultured with M-CSF (2.5 µg/mL) and FLL 7 d before being subjected to CCK-8 assay. (F) The monocytes were treated with M-CSF (2.5 µg/mL) and RANKL (5 µg/mL) for 7 d and assessed by tartrate-resistant acid phosphatase (Trap). FLL (1 µg/mL) significantly suppressed Trap activity. (*n* = 6). (G,H) Monocytes were treated with M-CSF (2.5 µg/mL) and RANKL (5 µg/mL) for 7 d and stained for Trap and F-actin rings. ^#^*p* < 0.05 compared with sham; ^##^*p* < 0.01 compared with sham; *p < 0.05 compared with OVX; **p < 0.01 compared with OVX (*n* = 3).

Trap activity and the level of Trap5b protein in plasma represent clinical osteoclast activity. Our study showed that Trap activity and Trap5b protein levels were significantly increased in the OVX group, and FLL treatment significantly suppressed these increases in OVX mice (*p*** **<** **0.01; Figure 5(A,B)). ACP5 and NFATc1 are two critical markers of osteoclastogenesis, and our results showed that mRNA expressions of ACP5 and NFATc1 were significantly higher in the OVX group than in the sham group. E_2_ and FLL then inhibited ACP5 and NFATc1 mRNA expression increases, respectively (*p*** **<** **0.05 or 0.01; Figure 5(C,D)). The effect of FLL on osteoclast differentiation and function was clarified by the isolation of monocytes from bone marrow. We observed that FLL significantly suppressed Trap activity without altering overall cellular activity as determined by the CCK-8 assay (Figure 5(E,F)). We also noticed that RANKL and M-CSF stimulated the formation of osteoclasts using Trap staining, whereas FLL inhibited osteoclast differentiation (Figure 5(G)). F-actin staining showed that FLL reduced the formation of F-actin rings (Figure 5(H)).

### Regulation of BMMSC differentiation by FLL

**Figure 6.**
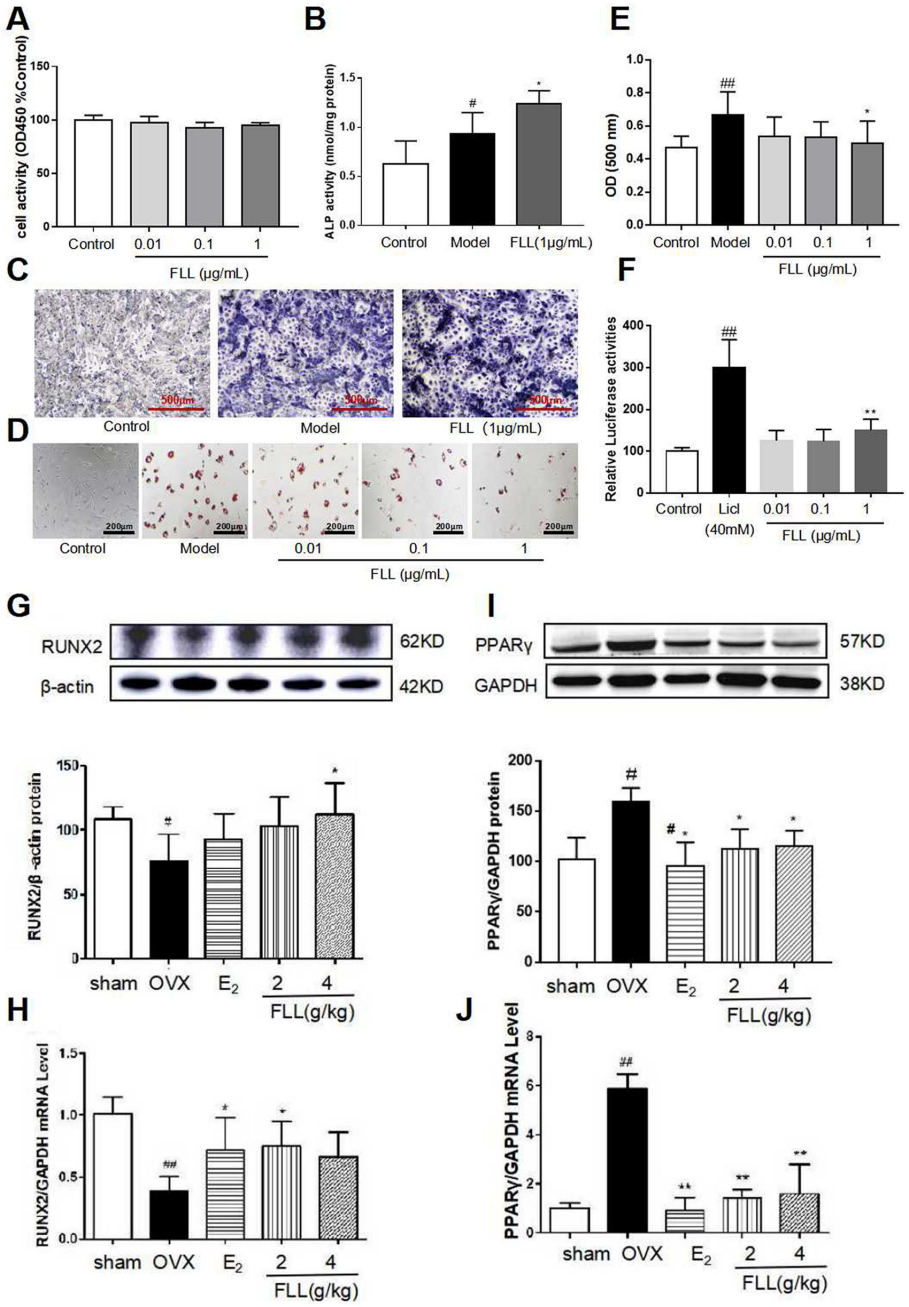
FLL significantly stimulates osteoblast differentiation and inhibits adipocyte differentiation of BMMSCs. (A) The BMMSCs were cultured with FLL and subjected to CCK-8 assay (*n* = 6). (B,C) BMMSCs were cultured with the osteogenic-inducer solution for 7 d and we then determined ALP activity, and ALP was also stained with BCIP/NBT; FLL markedly promoted the expression of ALP. (*n* = 6). (D) The BMMSCs were cultured with the adipocyte-inducer solution for 7 d and stained with Oil Red. (E) We quantified Oil Red by dissolving it in isopropanol and measuring the absorbance at 500 nm. FLL significantly inhibited adipocyte differentiation (*n* = 6). (F) TOPFlash assay was used to investigate the activation of the Wnt-signaling pathway by FLL, and results showed that FLL significantly activated Wnt signaling (*n* = 6). (G,H) The levels of RUNX2 protein and mRNA in mouse bone marrow were noticeably increased after treatment with FLL (*n* = 3). (I,J) The levels of PPARγ protein and mRNA in mouse bone marrow were notably decreased after treatment with FLL. (*n* = 3). #*p* < 0.05 compared with sham or control; ##*p* < 0.01 compared with sham or control; *p < 0.05 compared OVX or model; **p < 0.01 compared OVX or Licl.

BMMSCs induced osteoblast differentiation and adipocyte differentiation. Our results revealed that FLL significantly stimulated osteoblast differentiation and ALP production while inhibiting adipocyte differentiation and did not alter cellular activity as reflected by CCK8 (*p*** **<** **0.05; Figure 6(A–E)). We then used the TOPFlash assay to investigate the activation of the Wnt signaling pathway by FLL. The results showed that FLL significantly activated Wnt signaling (*p*** **<** **0.01; Figure 6(F)). As significant markers of BMMSC differentiation into osteoblasts and adipocytes, we determined the protein levels of RUNX2 and PPARγ in bone tissue by western immunoblotting analysis. We observed consistency in the effect of FLL on BMMSC differentiation, as FLL inhibited PPARγ expression but stimulated RUNX2 expression (*p*** **<** **0.01 or 0.05; Figure 6(G–J)).

### FLL inhibits fate determination and maturation of adipocytes

**Figure 7.**
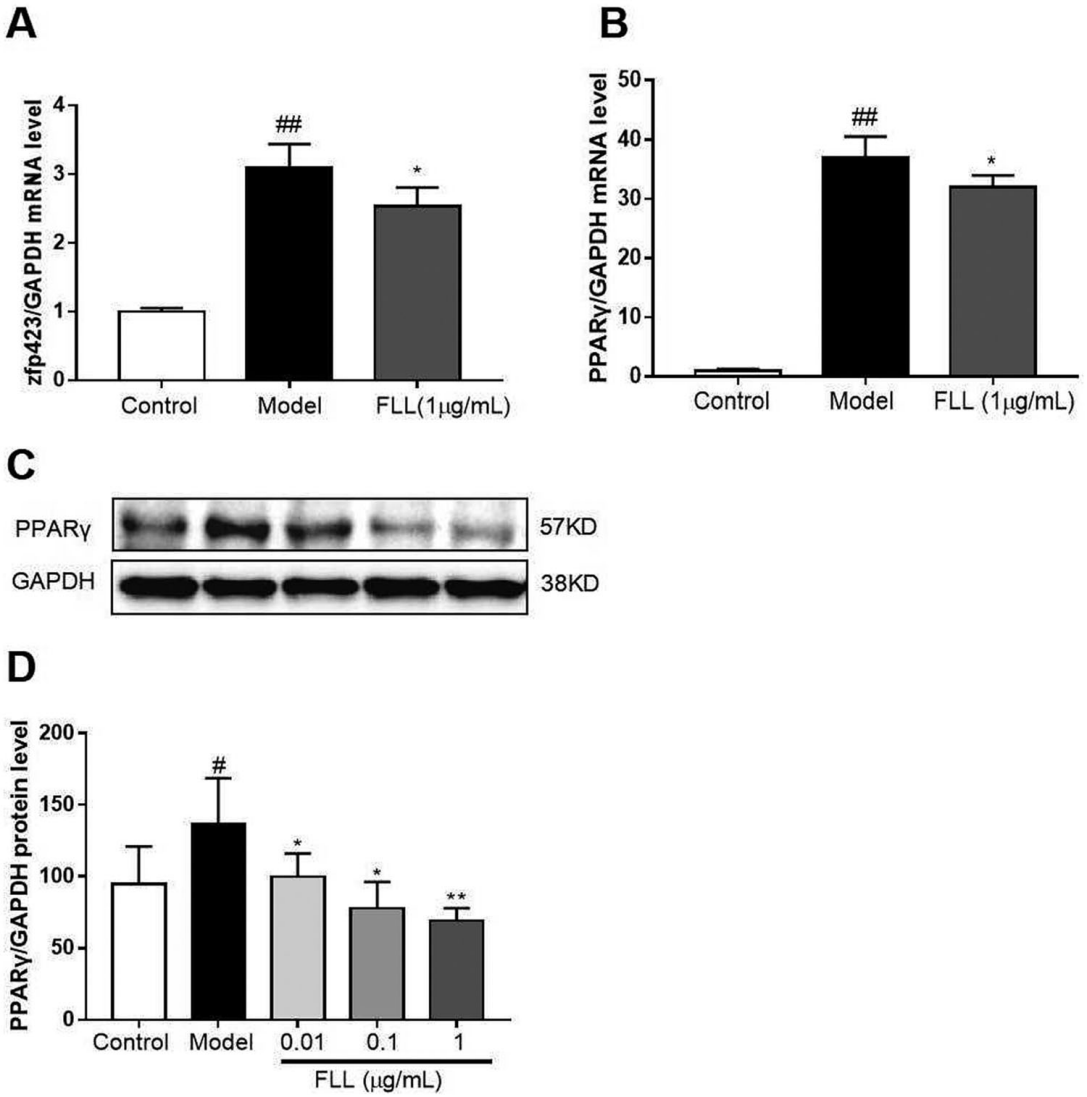
FLL inhibits the maturation of adipocytes but not of preadipocytes. (A) The BMMSCs were cultured with adipocyte-inducer solution for 1 d, and FLL inhibit zfp423 mRNA expression. (B–D) Incubation with FLL for 7 d significantly inhibited the level of PPARγ mRNA and protein. ^#^*p* < 0.05 compared with control; ^##^*p* < 0.01 compared with control; *p < 0.05 compared with the model; **p < 0.01 compared with the model (*n* = 3).

Zinc-finger protein 423 (Zfp423) is an important marker of preadipocytes, and we found that one-day incubation with adipocyte-inducer solution significantly increased Zfp423 mRNA expression (*p*** **<** **0.01; Figure 7(A)). PPARγ is a marker of mature adipocytes. When we evaluated the effect of FLL on PPARγ mRNA expression, we observed that incubation with FLL for seven days significantly inhibited the increase in PPARγ mRNA induced by the adipocyte-inducer solution (*p*** **<** **0.05; Figure 7(B–D)).

## Discussion

PMOP is a metabolic disorder characterized by decreased bone mineral density and increased fracture risk in postmenopausal women. Decreased circulating estrogen has been reported to be a significant cause of PMOP. OVX animals are used worldwide to investigate physiological and pathological changes in postmenopausal women, and OVX models are used in classical pharmacologic studies of PMOP (Chow et al. 2016). In modern medicine, current therapeutic principles for osteoporosis mainly rely on inhibiting bone resorption and promoting bone formation to bone mineral density. Clinical anti-osteoporosis drugs mainly include bisphosphonates, estrogenic hormones, parathyroid hormone peptides, calcium, and vitamin D (Fan et al. 2014; Hui et al. 2018; Xiao et al. 2018). However, modern medicine produces a high incidence of adverse reactions and complications, such as osteoradionecrosis of the jaws, atypical femoral fracture, stroke, cancer, and cardiovascular disease. In addition, modern medicines are typically expensive, which increases the medical burden on patients (Giuliani et al. 2005; Wang et al. 2012; Ruixian et al. 2017). Therefore, it is critical to adopt novel treatment methods to reduce the harm caused by osteoporosis. Traditional Chinese medicine does not have the concept of osteoporosis but calls it “bone atrophy” and “bone dryness”. Traditional Chinese medicine treatment of OP has gradually developed into an essential method with significant curative and low side effects. More recent studies have shown that most kidney-tonifying herbs have estrogen-like effects, which can then regulate bone metabolism, exerting anti-osteoporotic effects after menopause (Drake et al. 2017). Among the estrogen-like herbs, FLL is widely used to prevent and treat menopausal osteoporosis. It has been reported to increase estrogen levels in the body and provide estrogenic effects (Bonnet et al. 2017). In the present study, we used OVX mice to examine the effects of FLL on various organs. We found that FLL reversed OVX-induced uterine atrophy (Figure 1(B)). The present study showed that estradiol levels were not significantly increased after treatment with FLL in OVX mice. However, we found a higher trend than OVX mice. It indicates that the effect of FLL on PMOP may be independent of its estrogen-like effects (Figure 1(C)). It pushes us to find the exact mechanism in other areas.

Many researchers have found that menopausal women experience significant physiological changes, including a decrease in bone mass with an increase in visceral adiposity. These changes are major risk factors for osteoporosis, obesity, diabetes, and cardiovascular disease (Kase et al. 2020; Saleh et al. 2020); many studies have shown that estrogen deficiency is associated with bone loss and fat accumulation (Jang et al. 2020). The Women’s Health Initiative (WHI) shows that estrogen therapy increases BMD at multiple sites and reduces fracture risk by 24%. A systematic review reported a significant reduction in fracture risk with estrogen therapy compared with a placebo (Gartlehner et al. 2017). However, a decrease in circulating estrogen levels leads to a significant increase in circulating FSH levels; this may explain the prevalence of obesity in menopausal women (Liu et al. 2017). Therefore, osteoporosis and overweight due to increased adipose tissue are two crucial and common metabolic alterations in postmenopausal women. We found that FLL significantly reversed OVX-induced bone loss and increased fat mass in the present study. In contrast, FLL did not affect lean body mass, liver, spleen, or kidneys after administration of FLL for eight weeks (Figure 2). As body weight gain was significantly suppressed in the OVX group (Figure 2(A)), we measured the daily diet of each group (Table 2). Our results suggest that the suppression of body weight by FLL was independent of the daily diet. Therefore, our study may provide a new pharmacological treatment for menopause-related metabolic diseases such as PMOP and postmenopausal weight gain.

Bone remodeling is a complex process involving bone resorption and formation (Peyroteo et al. 2021; Yu et al. 2021). Therefore, the balance between bone resorption by osteoclasts and bone formation by osteoblasts is thus essential for maintaining proper bone metabolism and bone mineral density. It has been reported that osteoclasts are the only naturally occurring bone-resorbing cells in the body. It is characterized by large multinucleated cells derived from the monocyte/macrophage lineage. Increased numbers and activity of osteoclasts are responsible for bone destruction in abnormal metabolic bone diseases such as osteoporosis, osteoarthritis, and malignancies (Stopeck 2017; Duan et al. 2020; Yu et al. 2021). Therefore, inhibition of osteoclastogenesis may have a significant therapeutic effect in the treatment of osteoporosis. We demonstrate that FLL inhibits osteoclast activity induced by M-CSF and RANKL-induced osteoclast differentiation *in vitro*. After eight weeks of FLL treatment, Trap and Trap5b, two widely used markers of osteoclast activity (Rajfer et al. 2019), were significantly decreased in plasma, indicating inhibition of osteoclast activity. Furthermore, numerous studies have shown that the RANK/RANKL/OPG signaling pathway plays a crucial role in bone remodeling (Lyu et al. 2014; De et al. 2019). RANKL promotes the differentiation of osteoclast precursor cells into mature osteoclasts by binding to RANK in the cytoplasmic membrane (Ikebuchi et al. 2018); and OPG secreted by osteoblasts competitively binds to RANKL and blocks RANK/RANKL activation, thereby inhibiting the maturation and differentiation of osteoclasts (Liu et al. 2018). We also found that FLL increased OPG expression and decreased the expression of RANKL in plasma (Figure 3). Hence, the anti-osteoporotic effect of FLL may be achieved by inhibiting osteoclast differentiation through the RANK/RANKL/OPG signaling pathway. Furthermore, our research showed that treatment with FLL extract (1 µg/mL) significantly inhibited osteoclast differentiation and F-actin ring formation in mouse bone marrow monocytes (Figure 5). F-actin rings anchor to the mineralized matrix to form closed bone resorption compartments, a necessary structure for functional osteoclasts maturation (Li et al. 2017; Liu et al. 2020). Therefore, our study significantly inhibited the osteoclast activity of FLL both *in vitro* and *in vivo*.

Bone formation maintained by osteoblasts is another crucial aspect of bone remodeling. We propose that FLL inhibits adipocyte differentiation of BMMSCs and reduces abdominal fat in OVX mice by reducing the PPARγ/RUNX2 ratio (Figure 6). In that regard, the protective effect of FLL on bone metabolism was further enhanced.

As two common diseases occur in postmenopausal women, osteoporosis and obesity are major public health problems, and the relationship between PMOP and obesity has become an area of concern. There are many controversies in published studies on the effect of fat mass on bone tissue and associated fractures. Researchers believe that obesity has a long-term protective effect against osteoporosis and reduces the risk of fragility fractures due to a protective pad of fat mass during falls (Albala et al. 1996). However, there is growing evidence that the beneficial effects of fat mass on the whole body or bone are not the same for all postmenopausal women and depend upon overall fat distribution (Loh et al. 2019; Crivelli et al. 2021). Postmenopausal women are at increased risk of developing visceral obesity (Anagnostis et al. 2019; Dam et al. 2021), and visceral adipose tissue (VAT) is inversely associated with bone mass. There was a statistically significant inverse relationship between VAT and bone even after adjusting for age and sex (Zhang et al. 2015). In addition, high VAT and low estrogen negatively affect the skeleton in obese men (Ornstrup et al. 2015). Therefore, fat mass and bone may have a very close relationship. A previous study even showed that fat in the bone marrow might be responsible for osteoporosis (Wong et al. 2020). In our study, we also found that FLL significantly reversed the OVX-induced increase in bone marrow fat, which may constitute another mechanism for the anti-osteoporotic effect of FLL in the postmenopausal period.

There are two stages of adipogenic differentiation of BMMSCs. The first stage is the differentiation of preadipocytes, which are morphologically indistinguishable from BMMSCs but have lost the potential to differentiate into other mesenchymal lineages. The second stage is the terminal differentiation stage. Pre-adipocytes exhibit characteristics of mature adipocytes, i.e. lipid transport and synthesis capacity, insulin sensitivity, and secretion of adipokines (Liu et al. 2018). The protein zfp423 is a marker for pre-adipocytes, while PPARγ is a marker for mature adipocytes (Liu et al. 2015). Our study found that an expression of Zfp423 and PPARγ genes was increased in BMMSCs after one or seven days of induction with adipogenesis supplements. Furthermore, FLL significantly inhibited the expression of Zfp423 and PPARγ mRNAs (Figure 7), suggesting that FLL affects lineage fate determination and terminal differentiation by inhibiting adipogenic differentiation of BMMSCs.

## Conclusions

We demonstrate that FLL prevents estrogen deficiency-induced bone loss after eight weeks of use in adult female OVX mice. We found increased BMD, improved bone microarchitecture, inhibition of osteoclast activity, and decreased abdominal and bone marrow. Our findings also indicate that FLL restricts the activity and function of osteoclasts derived from bone marrow mononuclear macrophage cells, stimulates osteoblast differentiation, activates the Wnt signaling pathway, and inhibits adipocyte fate determination and maturation. Our results hint that long-term use of FLL may not produce any visible side effects. Our current and previous findings strongly suggest that FLL may be clinically valuable for treating menopause-related osteoporosis and weight gain but remains to be studied.
